# Proteomic profiling of bull spermatozoa and seminal plasma to inform the rational development of functionally targeted semen extenders for tropical cattle

**DOI:** 10.14202/vetworld.2026.1163-1177

**Published:** 2026-03-23

**Authors:** Sahiruddin Sahiruddin, Muhammad Yusuf, Athhar Manabi Diansyah, Masturi Masturi, Herdis Herdis, Tulus Maulana, Vinsensius Raymond Sihombing, Rahmat Rahmat, Muhammad Fajar Amrullah, Ahmad Alfaruqi Syahrandi Adam, Syahruddin Said, Andi Muhammad Alfian

**Affiliations:** 1 Faculty of Animal Science, Hasanuddin University, Makassar, South Sulawesi, Indonesia; 2 Research Center for Animal Husbandry, National Research and Innovation Agency (BRIN), Bogor, West Java, Indonesia; 3 Research Center for Applied Zoology, National Research and Innovation Agency (BRIN), Cibinong, West Java, Indonesia; 4 Faculty of Agriculture, Lambung Mangkurat University, Banjarbaru, South Kalimantan, Indonesia; 5 School of Veterinary Medicine and Biomedical Sciences, IPB University, Bogor, West Java, Indonesia

**Keywords:** Bali cattle, cryopreservation, proteomics, seminal plasma, semen extender, sperm motility, spermatozoa, tropical cattle

## Abstract

**Background and Aim:**

Artificial insemination (AI) is a cornerstone technology for genetic improvement in livestock; however, the fertility outcomes of cryopreserved semen often remain inconsistent, particularly in tropical production systems where heat stress and oxidative damage compromise sperm function. Conventional semen extenders are largely developed through empirical approaches and may not adequately reflect the molecular characteristics of locally adapted cattle breeds. Proteomic profiling offers an opportunity to identify endogenous proteins involved in sperm function and resilience, thereby enabling the rational design of functionally targeted semen extenders. This study aimed to characterize the proteomic profiles of bull spermatozoa and seminal plasma and to identify functional proteins associated with semen quality traits to inform the development of biologically informed extender formulations for tropical cattle.

**Materials and Methods:**

Semen samples were collected from three sexually mature Bali bulls maintained under standardized management conditions. Three ejaculates were obtained from each bull, resulting in nine ejaculates for evaluation. Semen quality parameters, including motility, viability, abnormality, acrosome integrity, and membrane integrity, were assessed using conventional microscopic techniques and computer-assisted sperm analysis. For proteomic analysis, spermatozoa and seminal plasma fractions were separated by centrifugation and subjected to protein extraction, enzymatic digestion, and high-resolution liquid chromatography–tandem mass spectrometry. Identified proteins were analyzed using bioinformatics tools for functional annotation, Gene Ontology classification, and protein–protein interaction analysis to determine their biological roles and potential relevance to semen preservation.

**Results:**

Fresh semen exhibited high motility (86.28% ± 3.26%), membrane integrity (86.35% ± 2.88%), and acrosome integrity (79.65% ± 6.93%), indicating overall favorable semen quality. Proteomic analysis identified 371 proteins, including 101 unique to spermatozoa and 270 shared between spermatozoa and seminal plasma. Functional annotation revealed that sperm proteins were predominantly associated with energy metabolism, cytoskeletal organization, and spermatogenesis, whereas seminal plasma proteins were enriched in antioxidant activity, immune response, and proteolytic processes. Key proteins involved in mitochondrial function, antioxidant defense, acrosomal activity, and structural integrity were identified and associated with semen quality parameters, including motility, viability, and membrane stability. Interaction network analysis further demonstrated coordinated relationships among mitochondrial enzymes, structural proteins, and fertilization-related molecules.

**Conclusion:**

The integration of semen quality assessment with proteomic profiling provides molecular insights into the biochemical environment supporting sperm function in Bali bulls. The identified proteins highlight critical pathways associated with energy production, oxidative stress protection, structural stability, and fertilization competence. These findings provide a conceptual framework for translating proteomic information into targeted additives for semen extenders, thereby supporting the development of biology-informed cryopreservation strategies tailored to tropical cattle production systems.

## INTRODUCTION

Artificial insemination (AI) plays a pivotal role in modern livestock breeding systems by enabling the efficient dissemination of superior genetics and accelerating herd improvement [1]. Despite its widespread adoption, AI outcomes often fall short of expectations, particularly in tropical production systems where pregnancy rates are often modest [2]. This discrepancy has drawn attention to a critical yet often underestimated factor in the reproductive chain: the quality and resilience of cryopreserved semen [3, 4]. These challenges are especially evident in tropical livestock systems, where many semen extender formulations are not specifically tailored to locally adapted breeds. During semen handling and cryopreservation, elevated thermal loads and oxidative stress can disrupt sperm membrane integrity, impair mitochondrial function, and destabilize cellular proteins. In tropical environments, chronic heat exposure and occasional cooling chain interruptions during semen handling may further intensify reactive oxygen species generation, destabilize mitochondrial membranes, and increase lipid peroxidation of the sperm plasma membrane. Collectively, these processes compromise membrane integrity, adenosine triphosphate (ATP) production, and flagellar activity, highlighting the need for extender formulations that provide adequate antioxidant protection and membrane-stabilizing components to mitigate heat- and osmolality-induced damage throughout semen processing, storage, and thawing.

Within this tropical context, Bali cattle represent an indigenous Indonesian breed characterized by remarkable heat tolerance, efficient utilization of low-quality forages, and resilience under smallholder production systems. These attributes make Bali bulls a biologically relevant model for studying semen quality and cryopreservation resilience in tropical environments. Despite their importance in regional livestock production, limited information is available regarding the molecular determinants underlying semen quality and cryopreservation tolerance in Bali bulls. Consequently, Bali bulls provide a valuable biological system for exploring the proteomic characteristics of semen associated with reproductive performance under tropical conditions. The proteomic signatures identified in this breed may reflect adaptive reproductive mechanisms in tropical environments, including chronic heat exposure and nutritional variability, thereby offering a biologically meaningful template for developing climate-resilient semen preservation strategies.

The cryopreservation process, although indispensable for long-term semen storage and genetic dissemination, exposes spermatozoa to multiple physical and biochemical stresses that can compromise structural integrity and functional competence [5, 6]. These alterations become particularly evident during the thawing phase, when damage to membranes, proteins, and metabolic pathways may become irreversible [7]. In this context, the semen extender, a protective medium designed to maintain sperm viability during storage and handling, plays a crucial role in determining post-thaw sperm quality [8, 9].

Traditional extenders derived from natural sources, such as egg yolk or milk, have long been used to mitigate cryodamage [10]. However, their empirical composition, biological variability, and potential microbial contamination present ongoing challenges [11, 12]. Moreover, many currently used formulations lack biological specificity and fail to account for molecular differences among breeds or environmental conditions. Most semen extender formulations have therefore been developed through empirical trial-and-error approaches, with limited incorporation of molecular insights into sperm physiology. This empirical strategy restricts the ability of extenders to be biologically tailored to breed-specific or environment-specific stressors. Consequently, many current semen extenders have been optimized without explicit consideration of the molecular pathways that support sperm survival, limiting their capacity to address breed-specific and environmental stressors. In contrast, proteomic profiling offers a promising approach to designing extenders aligned with endogenous proteins that regulate motility, membrane stability, and fertilization, thereby enabling a transition from empirically optimized formulations toward biology-informed cryopreservation systems.

Recent advances in proteomic technologies provide powerful tools for understanding sperm function at the molecular level [13]. Proteomics enables comprehensive characterization of the protein composition of spermatozoa and seminal plasma, revealing the roles of specific proteins involved in motility, membrane integrity, capacitation, and fertilization [14]. Such molecular insights allow researchers to identify endogenous biomolecules that naturally support sperm resilience, thereby guiding the rational development of functionally targeted semen extenders.

Although previous studies have investigated sperm proteomics in cattle, most have focused on identifying fertility biomarkers in commercial breeds raised under temperate production systems. Consequently, limited information is available regarding the molecular determinants of semen quality and cryopreservation resilience in indigenous tropical cattle such as Bali cattle. In addition, many earlier studies have analyzed either spermatozoa or seminal plasma independently, thereby overlooking the functional interplay between intracellular sperm proteins and extracellular seminal plasma components that collectively regulate sperm survival, motility, and fertilizing competence. This compartmental separation limits the ability to interpret the semen microenvironment as an integrated biological system. Furthermore, most studies have focused on descriptive protein cataloging or comparative differential expression analyses, without translating these molecular findings into practical applications for semen preservation. As a result, the development of semen extenders continues to rely largely on empirical formulations rather than on a mechanistic understanding of endogenous proteins that govern sperm physiology. This gap is particularly relevant in tropical production systems where chronic heat exposure, oxidative stress, and logistical challenges during semen handling may impose unique physiological pressures on sperm cells. Therefore, a comprehensive analysis that simultaneously characterizes the proteomic profiles of spermatozoa and seminal plasma in Bali bulls and interprets these molecular signatures in relation to semen quality traits remains lacking. Addressing this knowledge gap is essential for identifying biologically relevant proteins that support sperm resilience and translating these molecular insights into rational strategies to improve semen preservation under tropical conditions.

In this context, the present study aimed to characterize the proteomic profiles of bull spermatozoa and seminal plasma and to identify functional proteins associated with key semen quality parameters, including motility, viability, membrane integrity, and fertilization-related processes. By integrating semen quality evaluation with high-resolution proteomic analysis and bioinformatic functional annotation, this study sought to elucidate the molecular landscape underlying sperm function in Bali bulls. In addition, the study aimed to interpret the identified protein signatures in a translational framework by linking functional protein categories, such as antioxidant defense, mitochondrial energy metabolism, structural stability, and protein folding, with potential semen extender additives. Through this integrated approach, the study intends to provide a molecular foundation for the rational development of functionally targeted semen extenders that are aligned with the endogenous biochemical environment of bull semen and that may support more resilient cryopreservation strategies for tropical cattle production systems.

## MATERIALS AND METHODS

### Ethical approval

Semen samples were obtained from three 5-year-old Bali bulls that were sexually mature and reproductively active. All bulls had a body condition score ranging from 3.0 to 3.5 on a five-point scale, indicating optimal nutritional and physiological conditions for semen production. The animals were maintained under a standardized management system that included uniform housing and feeding practices at the Semen Processing Unit. Semen collection was carried out during the dry season (June–August) under tropical environmental conditions. Routine handling procedures were applied to minimize environmental and heat-related stress during semen collection. Three independent ejaculates were collected from each bull on separate days, resulting in a total of nine ejaculates used for semen quality evaluation and proteomic analysis. All procedures involving animals and biological sample handling were reviewed and approved by the Animal Use Ethics Committee for Research and Education, Faculty of Animal Science, Hasanuddin University (Approval No. 015/UN4.12/EC/VI/2025).

### Study period and location

This study was designed as an exploratory, discovery-oriented proteomic investigation intended to characterize the protein profiles of spermatozoa and seminal plasma. The study was conducted from June to August 2025 at the Animal Reproduction Laboratory, Semen Processing Unit, Faculty of Animal Science, Hasanuddin University, Makassar, Indonesia. Proteomic analyses were performed at the Center for Applied Zoology Research, National Research and Innovation Agency (BRIN), Cibinong, Indonesia.

### Semen collection and evaluation

Semen was collected from each bull using an artificial vagina (Minitube, Germany) following standard procedures. The same experienced technician performed all collections to ensure methodological consistency, and false mounts were not used during semen collection. Immediately after collection, semen samples were maintained at approximately body temperature and transported promptly to the laboratory for evaluation. All analyses described in this study were performed using fresh semen.

Fresh semen samples were subjected to routine macroscopic and microscopic evaluation before further analysis. Semen volume was recorded immediately after collection, and sperm concentration was determined using standard laboratory procedures. Only ejaculates that met the minimum quality criteria were included in subsequent semen evaluation and proteomic analysis.

Sperm motility was evaluated by placing 10 μL of fresh semen on a pre-warmed glass slide at 37°C and analyzing the sample using a computer-assisted sperm analysis (CASA) system (Vision v3.7.5, Minitube), following the method of Diansyah *et al*. [15]. The analysis was performed at a frame rate of 60 frames/s, which is commonly applied in commercial CASA systems for bovine sperm motility evaluation. CASA analysis was conducted using standardized software-defined settings in which sperm tracks were automatically filtered according to predefined criteria. Progressive motility was defined as sperm that exceeded the velocity and linearity thresholds established by the software. All CASA measurements were performed by a single trained operator, and multiple microscopic fields were evaluated for each sample to reduce analytical variability.

Sperm viability and morphological abnormalities were assessed by mixing 10 μL of semen with 10 μL of 2% eosin on a microscope slide. After air drying, the samples were examined using a trinocular microscope (Primo Star, Zeiss, Germany) connected to Indomicro View 3.7 software (Indomicro, Indonesia). Red-stained sperm cells were classified as non-viable, whereas unstained cells were considered viable. At least 200 sperm cells were evaluated per sample. Morphological abnormalities, including bent tails, detached heads, and irregular head shapes, were recorded. Acrosome integrity was evaluated by mixing semen with a formalin-saline solution (1% formalin in 0.9% sodium chloride [NaCl]) at a ratio of 1:4. Intact acrosomes were identified microscopically by the presence of a dark-stained tip on the sperm head. For membrane integrity assessment, 10 μL of semen was added to a hypo-osmotic swelling test solution prepared with 0.179 g NaCl in 100 mL of distilled water, and the mixture was incubated at 37°C for 30 min. Spermatozoa exhibiting curled tails were considered to possess intact membranes, whereas straight-tailed sperm were interpreted as having damaged membranes [16]. Each sample was analyzed across replicate microscopic fields.

### Proteomic analysis

Proteomic analysis was performed separately on the seminal plasma and spermatozoa fractions obtained from semen samples. Semen was centrifuged at 3,000 × g for 7 min to separate the supernatant (seminal plasma) from the spermatozoa pellet, and each fraction was processed independently using the same proteomic workflow. Each fraction was prepared from 0.5 mL of previously thawed frozen material at 37°C. Semen had previously been extended using a Tris–egg yolk-based extender containing glycerol and antibiotics during freezing. Before protein extraction, samples were washed three times with phosphate-buffered saline at 3,000 × *g* for 7 min to remove residual extender components. The final pellet or supernatant was then resuspended in 100 mM Tris-hydrochloride buffer (pH 7.9) containing 6 M urea.

The suspension was heated and sonicated for 2 h to ensure complete cellular lysis, after which cell debris was removed by centrifugation. Total protein concentration was determined using the bicinchoninic acid (BCA) assay (Thermo Fisher Scientific, Waltham, MA, USA) according to the manufacturer’s protocol. For in-solution digestion, 50 μg of protein was used per sample. Reduction was carried out with 100 mM dithiothreitol (DTT) at 36°C for 1 h, followed by alkylation with 200 mM iodoacetamide (IAA) at room temperature in the dark for 1 h. Sequence-grade modified trypsin (Promega, USA) was added at an enzyme-to-protein ratio of 1:50 (w/w), and proteins were digested overnight at 37°C. The reaction was terminated by adding 0.1% trifluoroacetic acid (TFA). The resulting peptides were desalted using C18 solid-phase extraction cartridges (Pierce C18 Spin Columns, Thermo Scientific), dried in a vacuum concentrator, and reconstituted in 0.1% formic acid before liquid chromatography–tandem mass spectrometry (LC–MS/MS) analysis.

Peptide mixtures were analyzed using a Thermo Scientific Vanquish Horizon ultra-high-performance liquid chromatography system coupled with an Orbitrap Exploris 240 high-resolution mass spectrometer (Thermo Fisher Scientific, Bremen, Germany). Chromatographic separation was carried out on an Acclaim PepMap 100 C18 column (150 mm × 1 mm, 3 μm) at 30°C with a flow rate of 75 μL/min. Mobile phases consisted of solvent A (mass spectrometry-grade water containing 0.1% formic acid) and solvent B (mass spectrometry-grade acetonitrile containing 0.1% formic acid). A gradient program was applied over 45 min: 5% solvent B for 1 min, a linear increase to 50% solvent B over 30 min, holding for 2 min, increasing to 90% solvent B over 2 min, and returning to 5% solvent B for the remainder of the run. The injection volume was 10 μL. Electrospray ionization was performed in positive mode with a spray voltage of 3.5 kV and a capillary temperature of 300°C, with sheath gas set to 35 units and auxiliary gas to 7 units. Data-dependent acquisition was conducted in top-20 mode, selecting the 20 most intense precursor ions from each full scan for MS² fragmentation, using an isolation window of 1.6 m/z, MS² resolution of 15,000 (at m/z 200), and a dynamic exclusion time of 30 s to prevent repeated sequencing of the same precursor ions.

Mass accuracy was maintained through routine external calibration of the Orbitrap analyzer, and no internal lock mass calibration was used. A pooled quality control sample was prepared by combining equal aliquots from all digested samples and injected at the beginning of the analytical run and after every ten injections. Blank injections containing 0.1% formic acid were inserted between selected samples to monitor carryover and instrument stability. Mass spectrometric data were acquired in full MS/data-dependent MS² mode with positive ionization, a resolution of 120,000 full width at half maximum (FWHM), a scan range of m/z 350–1200, a mass tolerance of 5 parts per million (ppm), and a normalized collision energy of 30 using nitrogen gas.

Raw spectra were analyzed using Proteome Discoverer version 2.5 software with the SequestHT search engine (Thermo Fisher Scientific), referencing the UniProt Bos taurus database for protein identification. A target–decoy strategy was used to control the false discovery rate (FDR), which was set at 1% at both the peptide and protein levels. Only peptides identified with high-confidence and containing at least two unique peptides per protein were retained for downstream analysis. Proteins meeting both the peptide identification and FDR criteria were considered confidently identified [15].

### Statistical analysis

Semen quality and kinematic parameters, including motility, viability, morphology, acrosome integrity, and membrane integrity, were summarized as mean ± standard deviation (SD) based on three biological replicates per bull. Approximate normality of the data distribution was assessed using the Shapiro–Wilk test together with visual inspection of Q–Q plots. The mean ± SD was therefore considered appropriate for describing central tendency and dispersion within this dataset. Descriptive statistical analyses were performed using the Statistical Package for the Social Sciences software, version 25.0 (IBM Corp., NY, USA).

**Figure 1:**
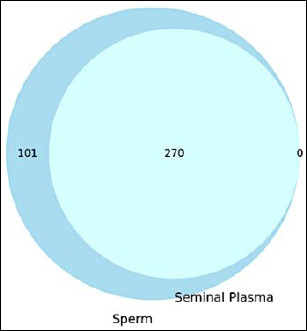
Venn diagram illustrating the distribution of identified proteins between sperm and seminal plasma fractions.

The proteomic component of this study was designed as an exploratory and primarily descriptive analysis focused on high-confidence protein identification and functional or pathway annotation rather than on quantitative differential proteomics or correlation modeling. Consequently, associations between identified proteins and semen traits are presented as biological interpretations supported by previous literature rather than as statistically tested quantitative relationships. Protein identification and quantification were performed using Proteome Discoverer version 2.5 software. Identified proteins were subsequently annotated using UniProt (Release 2025_04) for protein descriptions and molecular functions, PANTHER (Version 19.0, pantherdb.org) for gene ontology (GO) classification, and STRING (Version 12.0, string-db.org) for predicted protein–protein interaction networks and biological associations. No formal correlation analysis or regression modeling was performed between individual protein abundances and semen quality or kinematic parameters; therefore, no multiple-comparison correction was required. Any relationships between proteins and semen traits discussed in this study are based on functional annotation and previously published literature rather than on statistical association testing within this dataset. Figure 1 illustrates the schematic workflow of sperm evaluation, proteomic analysis, bioinformatic annotation, and translational mapping toward semen extender design.

## RESULTS

### Characteristics of semen quality

As summarized in Table 1, the semen samples used for proteomic analysis exhibited favorable values for motility, viability, membrane integrity, and acrosome integrity within the range typically reported for fertile bulls. The average sperm motility was 86.28 ± 3.26%, while membrane integrity reached 86.35 ± 2.88%, indicating strong vitality and preservation of structural stability. Sperm viability was recorded at 74.02 ± 6.90%, and acrosome integrity was 79.65 ± 6.93%, suggesting that the fertilization capacity of the spermatozoa was largely maintained. The abnormality rate remained relatively low at 19.52 ± 3.33%, indicating an overall healthy sperm population suitable for subsequent proteomic characterization.

**Table 1:** Semen quality parameters in bulls.

Parameter	Mean ± Standard deviation	95% Confidence interval
Motility (%)	86.28 ± 3.26	82.59 – 89.98
Abnormality (%)	19.52 ± 3.33	15.75 – 23.29
Viability (%)	74.02 ± 6.90	66.21 – 81.82
Acrosome integrity (%)	79.65 ± 6.93	71.81 – 87.49
Membrane integrity (%)	86.35 ± 2.88	83.09 – 89.60

Table 2 presents the kinematic characteristics of fresh semen, providing a detailed assessment of sperm motility beyond conventional observations. The mean curvilinear distance (DCL) of 51.66 ± 11.77 μm and curvilinear velocity (VCL) of 124.61 ± 29.55 μm/s reflect vigorous sperm movement along nonlinear trajectories, a feature essential for effective navigation within the female reproductive tract. The average path velocity (VAP) and straight-line velocity (VSL), recorded at 70.33 ± 19.03 μm/s and 51.19 ± 18.83 μm/s, respectively, indicate efficient forward progression toward the oocyte. The linearity (LIN) value of 41.56 ± 10.91% and straightness (STR) value of 72.42 ± 9.38% further demonstrate relatively directed and coordinated sperm movement. In addition, the wobble (WOB) value of 56.38 ± 8.15% describes the oscillation consistency of the sperm head, reflecting effective coordination between flagellar motion and forward progression.

**Table 2:** Sperm kinematic parameters in bulls.

Parameter	Mean ± Standard deviation	95% Confidence interval
DCL (μm)	51.66 ± 11.77	38.35 – 64.98
DAP (μm)	29.12 ± 8.20	19.84 – 38.39
DSL (μm)	21.13 ± 8.20	11.85 – 30.41
VCL (μm/s)	124.61 ± 29.55	91.17 – 158.04
VAP (μm/s)	70.33 ± 19.03	48.80 – 91.87
VSL (μm/s)	51.19 ± 18.83	29.89 – 72.50
LIN (%)	41.56 ± 10.91	29.21 – 53.91
STR (%)	72.42 ± 9.38	61.80 – 83.04
WOB (%)	56.38 ± 8.15	47.17 – 65.60

### Protein identification in spermatozoa and seminal plasma

**Figure 2:**
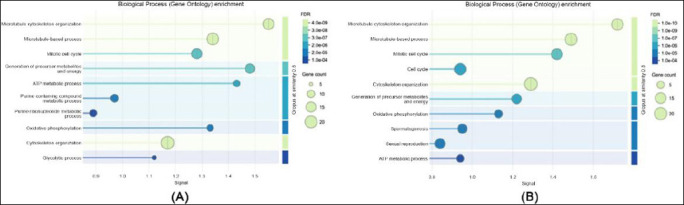
Gene ontology enrichment analysis of biological processes: (a) in sperm proteome and (b) in seminal plasma proteome.

Proteomic analysis identified a total of 371 unique proteins from spermatozoa and seminal plasma samples. As illustrated in Figure 2, 101 proteins were exclusively detected in the sperm fraction, whereas 270 proteins were shared between spermatozoa and seminal plasma. Interestingly, no proteins were uniquely detected in the seminal plasma fraction. This finding indicates a substantial proteomic overlap between the cellular and extracellular semen components, suggesting possible protein exchange mechanisms or a shared biological origin between the two fractions.

### Functional classification of identified proteins

GO enrichment analysis revealed clear functional distinctions between proteins detected in spermatozoa and those present in seminal plasma. Within the biological process category, sperm proteins were predominantly enriched in pathways associated with energy metabolism, protein folding, and spermatogenesis, whereas proteins identified in seminal plasma were mainly involved in immune response, proteolysis, and oxidative stress regulation.

**Figure 3:**
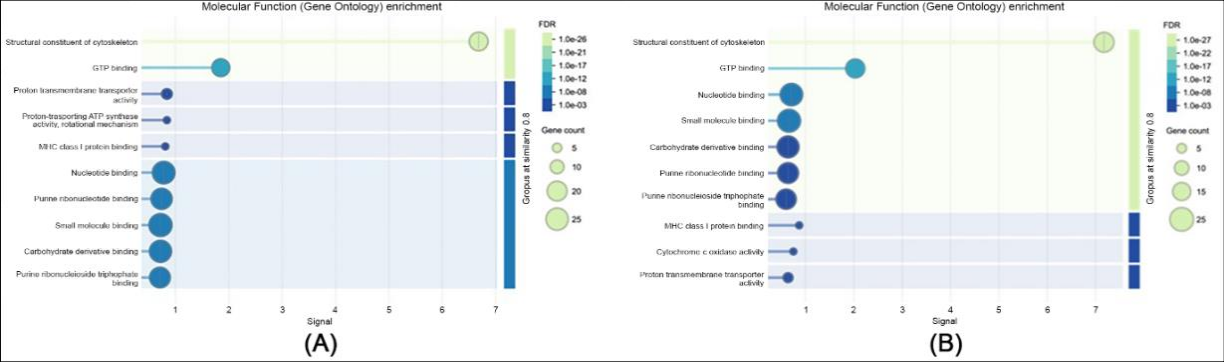
Gene ontology enrichment analysis of molecular functions: (a) in sperm proteome and (b) in seminal plasma proteome.

In terms of molecular function (Figure 3), sperm proteins were primarily associated with adenosine triphosphate (ATP) binding, structural molecule activity, and oxidoreductase functions, indicating their involvement in sperm motility and cellular energy metabolism. In contrast, seminal plasma proteins showed strong enrichment in peptidase activity, protein binding, and antioxidant functions, highlighting their role in protecting sperm cells and facilitating maturation processes.

**Figure 4:**
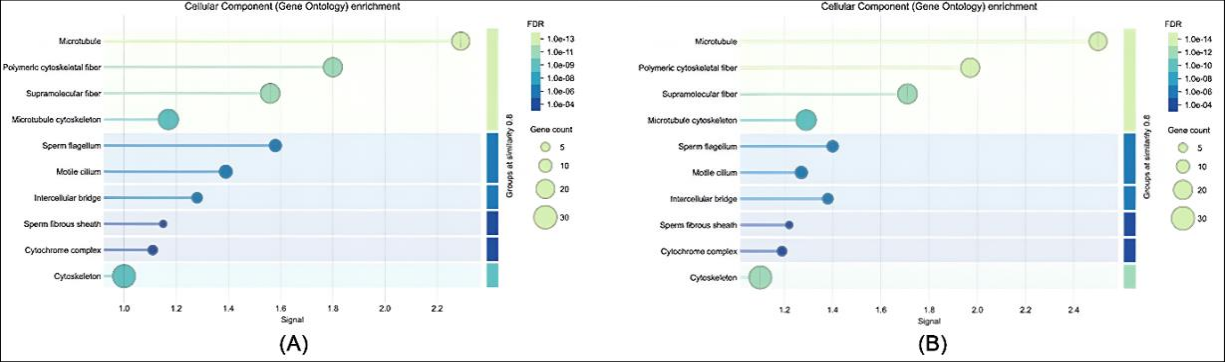
Gene ontology enrichment analysis of cellular components: (a) in sperm proteome and (b) in seminal plasma proteome.

Regarding cellular components (Figure 4), sperm proteins were mainly localized within mitochondria, flagellar structures, and acrosomal vesicles, reflecting their functional roles in motility and fertilization. Conversely, seminal plasma proteins were largely associated with extracellular regions, secretory vesicles, and exosomes, consistent with their involvement in extracellular signaling and protective functions. These results demonstrate a functional differentiation between intracellular sperm proteins and extracellular seminal plasma proteins and provide insight into their complementary roles in maintaining sperm viability and fertilizing ability.

### Functional proteins related to sperm function

Proteomic profiling of spermatozoa and seminal plasma revealed numerous functionally relevant proteins associated with critical semen parameters, as summarized in Supplementary Data (S1). These proteins were grouped into several functional categories, each contributing to sperm physiology and preservation.

Within the antioxidant and detoxification category, enzymes such as glutathione peroxidase 4 (GPX4) and saccharopine dehydrogenase-like oxidoreductases were identified. These proteins play protective roles against oxidative stress by neutralizing reactive oxygen species (ROS), thereby maintaining membrane stability and cellular viability during semen storage and processing.

The energy and mitochondrial category included multiple proteins associated with ATP generation and mitochondrial maintenance, including ATP synthase subunits, cytochrome c oxidase components, fumarate hydratase, and mitochondrial heat shock proteins. These proteins support sperm viability and motility by maintaining energy production and preserving mitochondrial functionality during cold shock and freeze–thaw stress.

Proteins associated with fertilization and acrosomal function were also detected, including members of the IZUMO family and acrosome-associated regulatory proteins. These molecules are involved in acrosome formation, sperm–egg membrane fusion, and fertilization competence. Their presence highlights the importance of preserving acrosomal integrity and enzymatic activity during semen preservation.

The motility and structural category contained abundant cytoskeletal proteins such as tubulin alpha and beta chains, dynein light chain, and several sperm microtubule inner proteins. These components form the core structure of the sperm flagellum and are essential for maintaining motility and directional movement, as reflected in kinematic parameters such as VSL, VCL, LIN, and STR.

Finally, proteins involved in protein folding and cellular stress responses were identified, particularly members of the heat shock protein family such as HSP60. These molecular chaperones assist in stabilizing and refolding denatured proteins during thermal or cryopreservation stress, thereby enhancing membrane stability and sperm survival.

### Protein interaction network

**Figure 5:**
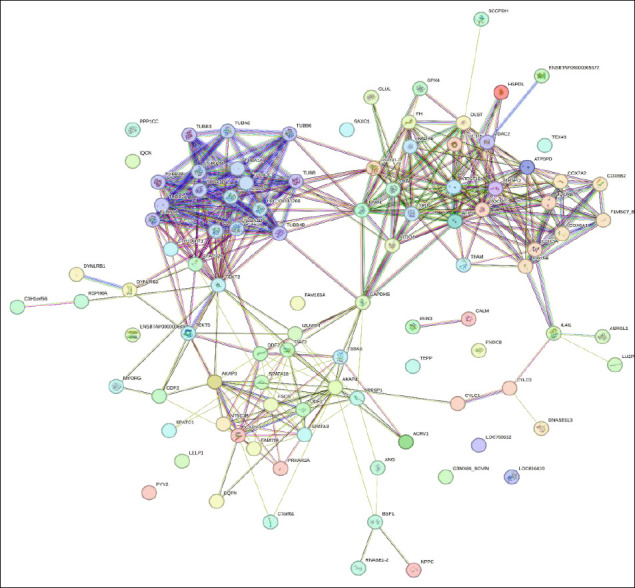
Protein–protein interaction network of identified proteins in sperm and seminal plasma.

Protein interaction network analysis of proteins detected in spermatozoa and seminal plasma revealed multiple interconnected clusters representing key biological functions (Figure 5). Tubulin alpha and beta chains belonging to the TUBA and TUBB families formed a prominent cluster with strong intranetwork connectivity, representing the structural backbone of the sperm flagellum responsible for motility.

Another dense cluster involved mitochondrial proteins, including ATP synthase subunits (ATP5F1A, ATP5F1B, and ATP5MC3) and cytochrome c oxidase components (COX5A, COX6B1, and UQCRC1). These proteins formed highly interconnected networks representing the energetic machinery that supports sperm viability and progressive motility.

Proteins related to fertilization, including IZUMO1, SPESP1, ACRV1, and acrosomal proteins, were also mapped within the interaction network and showed functional associations with signaling adaptor proteins such as AKAP4 and outer dense fiber proteins. These interactions suggest coordinated molecular mechanisms underlying acrosome reaction and sperm–egg fusion. The central positioning of AKAP3 and AKAP4 further emphasizes their role as scaffolding proteins that integrate structural and signaling pathways within the sperm cell.

Smaller peripheral subnetworks included antioxidant-related proteins such as peroxiredoxin 6 (PRDX6) and GPX4, together with stress response proteins, which exhibited targeted interactions with mitochondrial and metabolic regulators. Overall, this interaction network highlights the coordinated interplay among proteins involved in sperm motility, energy metabolism, structural integrity, and fertilization capability.

### Translational mapping of functional proteins to semen extender components

Functional annotation of the identified proteins enabled the targeted mapping of protein roles to potential semen extender components (Table 3) [17–27]. In this framework, candidate additives were ranked based on the consistency and relative abundance of their corresponding proteins across ejaculates, their network centrality within protein interaction clusters, and the functional importance of their protein category in relation to semen traits such as motility, membrane integrity, and acrosomal stability.

**Table 3:** Translational mapping of functional proteins to semen extender components.

Functional category	Candidate additive	Role of the additive in the extender	References
Antioxidant/Detoxification	Glutathione, Cysteine, N-acetylcysteine, Selenium nanoparticles, Curcumin, Quercetin, Selenium nanoparticles	Scavenging ROS, preserving membrane and DNA integrity	[17, 18]
Energy / mitochondrial	Fructose, pyruvate, glucose, creatine, coenzyme Q10, L-carnitine, MitoQ, resveratrol	Support ATP production and maintain mitochondrial activity	[19–21]
Fertilization/Acrosomal	Lecithin, soybean liposomes, cholesterol-loaded cyclodextrins, zinc ions, tetrahaloside	Stabilize acrosome and enhance fertilizing capability	[22–24]
Motility/Structural	Trehalose, sodium citrate, hydroxyethyl starch, Nanoclay, α-tubulin stabilizers (e.g., Taxol)	Preserving cytoskeletal structure and motility parameters	[19, 25]
Protein folding/stress response	HSP-mimetic peptides, tetrazolium, proline, chitosan nanoparticles, tumorosodeoxycholic acid	Preventing protein denaturation and aggregation under cryo-stress conditions	[26, 27]

Proteins involved in antioxidant and detoxification functions, including glutathione peroxidase and related oxidoreductases, guided the selection of antioxidant additives such as glutathione, cysteine, N-acetylcysteine, selenium nanoparticles, and phytochemicals, including curcumin and quercetin. These compounds are intended to mitigate oxidative stress and preserve membrane and DNA integrity during semen storage.

Proteins associated with energy metabolism and mitochondrial activity, including ATP synthase subunits, cytochrome c oxidase complexes, and fumarate hydratase, were linked to established extender additives, including fructose, glucose, pyruvate, creatine, L-carnitine, and coenzyme Q10, as well as mitochondria-targeted antioxidants such as MitoQ and resveratrol. These additives support ATP production and help maintain mitochondrial stability during semen preservation.

Structural and regulatory proteins related to fertilization, including IZUMO family members and acrosome-associated proteins, guided the selection of stabilizing agents such as lecithin, soybean liposomes, cholesterol-loaded cyclodextrins, zinc ions, and trehalose to maintain acrosomal integrity and enhance fertilization potential.

Proteins related to motility and cytoskeletal stability, including tubulin and axonemal components, were aligned with extender components, such as trehalose, sodium citrate, hydroxyethyl starch, and nanoclay, and with microtubule-stabilizing compounds, such as Taxol. These additives aim to preserve the cytoskeletal framework and maintain kinematic parameters following thawing.

Finally, proteins associated with protein folding and cellular stress responses, particularly heat shock proteins, were translated into protective extender additives, including HSP-mimetic peptides, proline, trehalose, chitosan nanoparticles, and tauroursodeoxycholic acid (TUDCA). These agents are intended to prevent cryo-induced protein misfolding and aggregation during semen cryopreservation.

Rather than proposing a generalized list of additives, this proteomic-to-extender mapping provides a hypothesis-generating framework that categorizes candidate components into near-term experimentally accessible formulations and longer-term exploratory strategies. Components in the first category are supported by abundant, central proteins associated with semen quality traits, whereas those in the latter category represent future directions that require dedicated toxicological evaluation and dose–response studies.

## DISCUSSION

### Proteomic framework for semen extender design

This study establishes a proteomic-informed framework for optimizing semen extender formulation by linking molecular findings with the rational selection of extender additives. Rather than functioning solely as a descriptive proteomic survey, the study uses the identified protein landscape to construct a conceptual engineering model in which functional protein groups are mapped to specific additive classes and their proposed cryoprotective roles. In this context, the inclusion of fresh semen quality data (Table 1) and detailed sperm kinematic parameters (Table 2) is not merely descriptive but essential for interpreting the downstream proteomic landscape. These phenotypic traits serve as biological indicators of underlying protein integrity and function, thereby allowing a more biologically grounded interpretation of sperm resilience. The integration of semen phenotype and proteomic data provides a more comprehensive understanding of sperm preservation biology and supports the development of targeted additive strategies. In addition, the simultaneous analysis of spermatozoa and seminal plasma offers a system-level view of the reproductive microenvironment by showing how intracellular machinery and extracellular protective factors act in complementary and partially overlapping ways that can be exploited in extender design.

### Structural proteins and sperm kinematics

The elevated values of straight-line velocity (VSL), curvilinear velocity (VCL), and average path velocity (VAP) indicate the presence of a structurally intact and metabolically efficient flagellar apparatus [28]. This interpretation is supported by the abundant detection of axonemal and cytoskeletal proteins, including tubulin isoforms, dyneins, and other microtubule-associated components, as shown in Supplementary Data (S1). These findings support the inclusion of structural stabilizers such as trehalose or alpha-tubulin-stabilizing agents, including Taxol analogs, to preserve microtubule integrity during freeze–thaw stress, as previously suggested by Sharafi *et al*. [28] and Qi *et al*. [29].

### Mitochondrial proteins and energy preservation

The marked expression of mitochondrial proteins, including ATP synthase subunits, cytochrome c oxidase complexes, and tricarboxylic acid cycle enzymes, confirms the dependence of sperm motility and viability on oxidative phosphorylation [29, 30]. These proteins reflect both high energetic demand and susceptibility to oxidative and osmotic stress. Previous studies have shown that supplementation with L-carnitine, pyruvate, and coenzyme Q10 can improve mitochondrial preservation and post-thaw recovery [31–33], supporting their inclusion in functionally tailored extenders. The predominance of mitochondrial and antioxidant proteins in Bali bull semen is consistent with the possibility that reproductive function in this breed has adapted to sustained thermal load and redox challenge, further emphasizing its relevance as a model for semen preservation strategies under tropical conditions.

### Acrosomal proteins and fertilization competence

The identification of IZUMO family members and acrosomal assembly-related proteins emphasizes the importance of preserving acrosomal integrity at the fertilization interface. Because these proteins mediate oocyte fusion [34], their susceptibility to cryo-induced membrane and lipid damage supports the use of membrane-stabilizing additives such as lecithin, cyclodextrin-complexed cholesterol, and zinc. These components have previously been reported to improve acrosomal enzyme retention and support post-thaw fertility [29, 35]. Therefore, the preservation of acrosomal structure should be considered a central goal in extender formulation.

### Antioxidant proteins and redox regulation

The seminal plasma proteome further supports the sperm findings, particularly with respect to oxidative stress regulation. The enrichment of glutathione peroxidase 4 (GPX4), thioredoxin, and peroxiredoxins indicates that bull semen exists in a redox-sensitive environment in which antioxidant defense is crucial for sperm survival [36, 37]. This observation supports the incorporation of both conventional antioxidants, such as glutathione and N-acetylcysteine, and more advanced delivery systems, including selenium nanoparticles and curcumin-loaded liposomes, to enhance ROS scavenging efficiency and improve bioavailability [38].

### Interpretation of the absence of seminal plasma-specific proteins

A noteworthy observation in this study was the absence of proteins unique to the seminal plasma fraction. From a biological perspective, this pattern may reflect the close molecular interaction between spermatozoa and the surrounding seminal fluid, because proteins secreted by accessory sex glands may adsorb to the sperm surface or be distributed across both cellular and acellular compartments. However, technical factors should also be considered. These may include the extraction protocol, the relative protein load in spermatozoa and seminal plasma fractions, the detection sensitivity of the liquid chromatography–tandem mass spectrometry platform, and database filtering criteria, all of which may have limited the detection of low-abundance proteins specific to seminal plasma. Accordingly, the absence of seminal plasma-specific proteins should be interpreted with caution, and future studies employing optimized workflows for low-abundance secreted proteins are warranted.

### Stress response proteins and cryoprotection

The detection of multiple heat shock proteins, including heat shock protein 60 (HSP60), and other chaperonins indicates cellular stress during cryopreservation. These proteins play central roles in protein folding, stabilization, and protection against stress-induced denaturation [39]. Their presence supports the use of extender additives such as HSP-mimetic peptides, trehalose, and tauroursodeoxycholic acid (TUDCA), which may help preserve protein conformation and reduce structural damage during cryogenic stress [40].

### Functional protein groups and translational relevance

Supplementary Data (S1) further demonstrate that the identified proteins can be grouped into antioxidant, mitochondrial, acrosomal, structural, and stress response classes, each of which is linked to specific semen quality traits. This classification bridges molecular function with phenotypic expression. For example, the co-occurrence of mitochondrial proteins with high VAP and curvilinear distance (DCL) values indicates active ATP generation and highlights the importance of including energy substrates such as fructose and pyruvate in extender formulations [30]. Similarly, the presence of proteins involved in cytoskeletal organization directly supports the observed robustness of sperm kinematics and strengthens the rationale for including polymer-stabilizing compounds.

### Protein interaction network and additive prioritization

The protein interaction network (Figure 5) revealed central nodes such as ATP synthase and tubulin that connect multiple functional pathways. Targeting such central protein complexes may provide broader protective benefits during cryopreservation than focusing on isolated pathways alone. In addition, this network structure helps identify less well-characterized interacting proteins that may represent novel candidates for future extender development. Thus, the interaction network serves not only as a descriptive tool but also as a framework for prioritizing additive selection based on functional centrality and pathway integration.

### Translational mapping toward next-generation extenders

Table 3 translates these proteomic signatures into practical additive recommendations and provides a targeted strategy for semen extender design. This translational mapping combines established extender components with innovative bioengineered options, including nanoparticle-based delivery systems, proline-loaded carriers, and bioactive liposomal formulations. Such nanotechnological platforms offer the potential for precision targeting, sustained release, and synergistic action, thereby representing a shift from empirical semen preservation toward evidence-guided and mechanism-based formulation strategies [41, 42].

### Biological significance of phenotype–proteome alignment

The close alignment between semen phenotypes and proteomic signatures strengthens the biological rationale for component selection. Elevated expression of mitochondrial proteins parallels improved kinematic values, whereas the presence of fusion-related proteins supports the inclusion of acrosomal protectants [43]. This systems-level integration addresses the multifactorial nature of cryodamage and supports the development of breed- or species-specific extenders that more closely reflect the biological requirements of sperm cells.

### Study limitations

These findings should be interpreted within an exploratory, hypothesis-generating framework, as the present study did not employ a dedicated quantitative differential proteomics workflow. Therefore, future quantitative studies are needed to confirm subtle differences in individual protein abundance. In addition, several limitations should be acknowledged. First, proteomic profiles reflect protein presence and relative expression rather than direct functional activity under cryo-stress. Second, the proposed additive–protein relationships remain conceptual and require empirical validation, including dose optimization and assessment of possible antagonistic effects among additives. Third, post-translational modifications, which may critically influence protein function, were not assessed in the present study and should be investigated in future work.

### Future perspectives

Future studies should validate these proteomic-informed extender concepts through standardized post-thaw semen assessments and controlled fertility trials to translate the current conceptual framework into robust, field-applicable formulations (Figure 5). Bioinformatics-guided nanoparticle engineering also represents a promising direction for developing next-generation extender systems with improved specificity and biocompatibility. Overall, this study proposes a proteomics-to-formulation pipeline that conceptually links molecular findings to candidate extender components. Although its practical effectiveness remains to be confirmed through post-thaw and fertility studies, the framework advances a precision cryobiology approach in which extender composition is explicitly aligned with species-, breed-, and function-specific proteomic signatures. In this way, formulation strategies are guided by the molecular requirements of sperm cells themselves rather than being determined secondarily by empirical convention. Such an approach may have scalable applications not only in livestock reproduction, but also in endangered species conservation and human-assisted reproductive technologies.

## CONCLUSION

This study integrates semen quality evaluation, sperm kinematic analysis, and proteomic profiling to elucidate the molecular landscape underlying sperm function in Bali bulls and to inform the rational design of semen extenders. Fresh semen exhibited favorable reproductive characteristics, including high motility (86.28 ± 3.26%), membrane integrity (86.35 ± 2.88%), and acrosome integrity (79.65 ± 6.93%), indicating that the ejaculates used for proteomic analysis represented functionally competent sperm populations. Proteomic analysis identified 371 proteins across spermatozoa and seminal plasma, with 101 proteins specific to spermatozoa and 270 shared between the two compartments. Functional annotation revealed that these proteins were primarily associated with antioxidant defense, mitochondrial energy metabolism, cytoskeletal organization, acrosomal function, and protein folding processes. Interaction network analysis further highlighted key molecular hubs, including tubulin and adenosine triphosphate synthase complexes, that coordinate pathways involved in sperm motility, structural integrity, and fertilization capacity.

The findings provide practical implications for semen preservation by demonstrating how specific functional protein groups can be translated into targeted semen extender components. Antioxidant-related proteins support the inclusion of ROS scavengers such as glutathione and N-acetylcysteine, whereas mitochondrial enzymes highlight the importance of energy-supporting substrates such as fructose, pyruvate, and L-carnitine. Structural proteins associated with flagellar architecture indicate the need for cytoskeletal stabilizers such as trehalose, while proteins involved in acrosomal formation and sperm–egg fusion emphasize the importance of membrane-stabilizing agents such as lecithin, cholesterol-loaded cyclodextrins, and zinc. These insights demonstrate that extender formulation can be guided by molecular evidence rather than relying solely on empirical approaches.

A key strength of this study lies in the integrated analysis of semen phenotypes and proteomic signatures, combined with the simultaneous characterization of spermatozoa and seminal plasma. This dual-compartment approach provides a system-level perspective on the reproductive microenvironment and reveals how intracellular and extracellular proteins jointly support sperm survival and function. By linking functional protein categories with semen traits and extender additives, the study establishes a conceptual proteomics-to-formulation pipeline that bridges molecular discovery with practical applications in cryopreservation.

Overall, the results highlight the potential of proteomic approaches to support precision cryobiology strategies in which semen extender composition is aligned with the endogenous biochemical environment of sperm cells. Although the proposed additive mapping requires experimental validation through post-thaw assessments and fertility trials, the framework developed in this study provides a biologically informed foundation for designing next-generation semen extenders tailored to tropical cattle production systems. Such approaches may ultimately improve reproductive efficiency in livestock breeding programs while also offering broader applications in animal conservation and assisted reproductive technologies.

## DATA AVAILABILITY

The supplementary data can be made available from the corresponding author upon request.

## AUTHORS’ CONTRIBUTIONS

SaS: Resources, project leadership of the study, and writing of the original draft. MY: Conceptualization and writing, review, and editing. AMD: Formal analysis, methodological analysis, and writing, review, and editing. MM: Field sampling supervision and writing, review, and editing. HH: Statistical analysis and writing, review, and editing. TM: Validation and writing, review, and editing. VRS: Assisted with sample collection and processing. RR: Statistical analysis and writing, review, and editing. MFA: Assisted with sample collection and methodological analysis. AASA: Data curation and writing, review, and editing. SyS: Validation and writing, review, and editing. AMA: Sample processing, software, and writing, review, and editing. All authors read and approved the final version of the manuscript.
